# Evaluation of In Vitro Antioxidant, Anti-Obesity, and Anti-Diabetic Activities of *Opuntia ficus* Cladodes Gel and Its Application as a Preservative Coating for Shrimp during Refrigerated Storage

**DOI:** 10.3390/gels9090716

**Published:** 2023-09-04

**Authors:** Alaa S. Mohamed, Essam Mohamed Elsebaie, Wesam Mohammed Abdelrhman, Nabila Yahia Mahmoud Abdulmaguid, Rasha M. Bahnasy, Manal Salah Abbas Elgendy, Arwa Mohamed Mohamed Mahmoud Elashry, Marwa Fawzy El-Hassanin, Nora Hamdy Mouhamed El-Wakeil, Azhar Mostafa Mohamed Khalil, Hesham F. Amin

**Affiliations:** 1 Food Science Department, Faculty of Agriculture, Zagazig University, Zagazig 44511, Egypt; 2 Food Technology Department, Faculty of Agriculture, Kafrelsheikh University, Kafr El-Shaikh 33516, Egypt; 3 Nutrition & Food Science Department, Faculty of Home Economics, Al-Azhar University, Tanta 31512, Egypt; 4 Food Science and Nutrition Department, Science Collage, Taif University, P.O. Box 11099, Taif 21944, Saudi Arabia; 5 Food Science & Technology Department, Faculty of Home Economics, Al-Azhar University, Tanta 31512, Egypt; 6 Department of Fish Processing and Technology, Faculty of Fish Resources, Suez University, Suez 43511, Egypt

**Keywords:** DPPH, ABTS, OFC gel, TBA, shrimp, lipase

## Abstract

*Opuntia ficus* cladodes (OFC) are considered one of the wastes that result from *opuntia* cultivation, and their disposal by traditional methods results in many environmental problems. Therefore, this study was conducted with two aims. The first was the production of OFC gel, and the evaluation of its in vitro antioxidant (by two methods, DPPH and ABTS), anti-obesity, and anti-diabetic activities. The second was an investigation of the effects of different concentrations of this gel (0, 50, and 100%) as an edible coating on the quality of shrimp during 8 days of refrigerated storage. The results showed that this gel was characterised by a high content of ash (10.42%), total carbohydrates (75.17%), and total phenols (19.79 mg GAE/g). OFC gel contained six types of sugars: arabinose, xylose, galactose, rhamnose, glucose, and uronic acid, and the most abundant was xylose (36.72%). It is also clear from the results that the OFC gel had high antioxidant properties, which were higher against DPPH than ABTS at the same concentration. OFC gel showed a high inhibition activity against lipase, α-glycosidase, and α-amylase enzymes, and their IC_50_ values were 1.43 mg/mL, 0.78 mg/mL, and 0.57 mg/mL, respectively. The results also stated that shrimp coated with OFC gel had lower pH, drip loss, TVB-N, and TBA values through the days of refrigerated storage. Moreover, the shrimp coated with 100% OFC gel were better than those coated with 50% OFC gel. In conclusion, OFC gel showed high potency as active antioxidant, for its enzyme anti-activities, and as an edible coating for shrimp.

## 1. Introduction

*Opuntia ficus indica* (L.) (OFI) refers to a succulent, spiky, perennial plant that belongs to the Cactaceae family. It is also referred to as a prickly pear or nopal cactus around the world [1]. The Opuntia genus has over 1500 cactus species, the most common of which is OFI [2]. OFI is a significant crop in dry and semi-arid locations across the world [3]. It has been grown for many decades in Egypt’s sandy soils [4].

The fruit is still the principal economic product of this plant, despite the fact that the mature plant of OFI has a median yield of 250 cladodes, resulting in 625 kg/plant, rendering this plant-based item readily available [5]. Cladodes are commonly utilised as feed for animals [6], fodder, or are disposed of under landfills, but in some countries they can be used as human food [7].

Cladodes are high in polyphenols, but they also have polysaccharides and soluble fibres, which can help with hyperglycemia and other physiological problems. OFI’s health advantages have recently attracted a lot of attention due to its high concentration of bioactive components [8].

OFI gel is a type of polymer found in the cells of the parenchyma of tissues that plays an important function in water preservation. Cladodes have a higher gel content than the rest of the plant portions. Compared to adult cladodes, which include more fibres and ashes, young cladodes are more abundant in phenolic components, proteins, and water [9]. OFI mucilage, also known as gel, is a hetero-polysaccharide with a high molecular weight that mostly consists of six sugars (galactose, arabinose, xylose, rhamnose, galacturonic acid, and glucose), biomolecules, and proteins [10]. The gel generated from the OFI cladodes induced hypoglycemia and elevated insulin levels in the plasma of rats [11]. Kalegowda et al. [12] also stated that the gel has in vitro antioxidant and anti-hyperlipidemia properties.

Obesity and hyperglycemia are two of the physiological disturbances that characterise metabolic syndrome. This is a big and growing health and clinical concern across the world [13]. Diabetes has grown to be a serious global health issue. It is a metabolic condition with high levels of glucose in the blood that may also lead to other health issues, including neuropathy, heart disease, weakness, high blood pressure, gangrene, nephropathy, retinopathy and other disorders [14]. Inhibiting the digestive enzymes, namely α-glucosidase and α-amylase, is one of the treatment strategies intended to reduce the glucose generation from the digestion of carbohydrates. Acarbose has been utilised in clinical studies as an efficient inhibitor of carbohydrate hydrolysis; nevertheless, it has certain adverse effects, such as stomach discomfort, flatulence, and diarrhoea [15].

Obesity is thought to increase the risk of developing type 2 diabetes mellitus, stroke, cardiovascular disease, cancer, and hypertension [16]. Obesity is caused by high-fat and high-calorie diets, as well as a lack of activity [17]. The small intestine’s ability to absorb triacylglycerol is linked to obesity and disorders that are related to it. Medications can help obese people lose weight through diet management. When dietary fat is exposed to the activity of pancreatic lipases, which hydrolyze tri-acylglycerols to mono-acylglycerols and fatty acids, it is absorbed by the gut [18]. Consequently, inhibiting pancreatic lipase might be a significant way to control obesity. Orlistat, a gastro–pancreatic lipase inhibitory agent, is now licenced for the treatment of long-term obesity, although it produces gastrointestinal adverse effects such as oily spotting, bloating, faecal urgency, faecal incontinence, and steatorrhoea [19].

Shrimp is a popular seafood with significant nutritional and economic value in many parts of the world [20]. However, it is perishable because of the activities of enzymes and microorganisms that, while stored, result in lipid oxidation, black melanin pigments, disagreeable odour and taste, and tissue degradation [21]. Conventional preservation techniques, such as chilling, are ineffective, and chemical additives such as sulphates and their derived compounds have recently been employed to extend the shrimp’s shelf life [22]. Because of the allergy symptoms of these chemicals and the increased need for healthy meals, researchers are looking for alternate strategies to reduce the residual sulphite content of shrimp [23]. Irradiation [24], high hydrostatic pressure [25], and cold plasma [26] have all been employed for preserving seafood. Regardless of their effectiveness, these procedures are not reasonably priced. The application of edible coatings and films, either alone or in combination with natural preservatives, is a feasible and readily accessible strategy for extending the shelf life of fresh foods [27]. 

Salehi et al. [28] have presented several contemporary food industry applications of OFI gel, which include its use as an edible film and coating to improve agricultural products shelf life. There is no information about studying the in vitro antioxidant, anti-obesity and anti-diabetic activities of the opuntia ficus cladodes (OFC) gel or using it as an edible coating for shrimp. Consequently, the goal of this work was to examine the in vitro antioxidant, anti-obesity and anti-diabetic activities of OFC gel as well as to investigate the effects of different concentrations of OPC gel (0, 50 and 100%) on the quality of shrimp during refrigerated storage.

## 2. Results and Discussion

### 2.1. Proximate Analysis

Table 1 shows the findings from measuring the proximate chemical composition of the OFC gel based on dry weight. The moisture level (7.98%) is below the pharmacopoeial level established for natural mucilages (gels) and gums (≤15.0%) [29]. OFC gel has a moisture content of 7.98%, which is equivalent to the 7.62% reported by Rodríguez-González et al. [30]. The ether extract had a minimal concentration with a percentage of 0.12%, which agreed with the comparable research of Rodríguez-González et al. [30], which revealed that the percentage of fat in dried gel prepared from the six species of *Opuntia* cladodes ranged from 0.06% to 0.2%. Lipids, on the other hand, are fundamental chemicals in plants that are necessary for life and growth due to the fact that they act an essential function in energy storage; nevertheless, they are only stored in specialised tissues and cells and are seldom stored in leaves, roots, and stems [31].

The OFC gel protein concentration was 6.74%, according to data in the same table. The obtained values were higher than those estimated by Du Toit et al. [32], as well as inside the range reported in previous studies [30,33], which showed that the protein content of dried OFC gel samples ranged from 4.01 to 8.26%. The ash percentage in OFC gel produced in this investigation (10.42%) was close to that published by Rodríguez-González et al. [30], who recorded 11.91%; however, our findings contrast with those reported by Sepúlveda et al. [34], who stated a mean percentage of 37.3%. However, the crude fibre content noticed in OFC gel (0.46%) was lower than that reported by Sepúlveda et al. [34] (0.69%) but greater than that obtained by Du Toit et al. [32] and Gebresamuel and Gebre-Mariam [33] (0.2 and 0.06%, respectively).

OFC gel is thought to be made up of three water-soluble fractions: (1) a non-gelling mucilage; (2) a pectin with calcium-dependent gelling abilities; and (3) a polysaccharide fraction with thickening attributes, which comprises no more than 10% of the water-soluble substance [35]. Because only water-insoluble components, such as lignin, hemicellulose, and cellulose, are assessed in crude fibre analysis, a low crude fibre content was predicted [36]. According to the data in Table 1, the OFC gel total carbohydrate content was 75.17%, which is consistent with de Andrade Vieira et al. [37] who indicated that the OFC gel contained carbohydrate ranging from 39.77% to 87.45%. Total phenolic, total flavonoids, and total flavonol contents were 19.79 mg GAE/g, 9.05 mg QE/g, and 2.54 mg QE/g, respectively, according to the data in Table 1. Our findings were similar to those published by Procacci et al. [38], but somewhat lower than those given by Gheribi et al. [39]. Furthermore, the same table data indicated that OFC gel had a sub-acidic pH (4.58). The OFC gel pH value was less than that revealed by Kalegowda et al. [12] for Opuntia dillenii cladode gel (7) but equivalent to that provided by Procacci et al. [38].

### 2.2. Sugars Identification for OFC Gel

The monosaccharide content of OFC gel is shown in Table 2. Table 2 reveals the presence of six sugars in OFC gel: arabinose, xylose, galactose, rhamnose, glucose, and uronic acid. The results indicated that xylose is the most abundant sugar in OFC gel, followed by uronic acid. Our findings are consistent with those of Majdoub et al. [35] and Ribeiro et al. [40]. The most prevalent sugars found in the OFC gel (in ascending order, Table 2) were arabinose (3.82%), glucose (4.62%), rhamnose (12.57%), galactose (13.69%), uronic acid (28.35%), and xylose (36.72%).

The obtained results were comparable to those recorded by Elshewy et al. [41], but they differ from those presented by Kalegowda et al. [12], Rodríguez-González et al. [30], and de Andrade Vieira et al. [37]. According to Otálora et al. [10], the variations in OFC gel sugar composition indicated by numerous researchers could differ, based on the extraction procedure, location, and additional environmental variables, including climate, the cladode’s age, and the soil in which plants grow. Galacturonic acid content in OFC gel, in particular, plays an important role in its ability to form a film, including the viscosity, water-holding ability, and ability of chelation, emphasising the various possible uses of our extracted gel.

### 2.3. OFC Gel Antioxidant Activity

**Figure 1:**
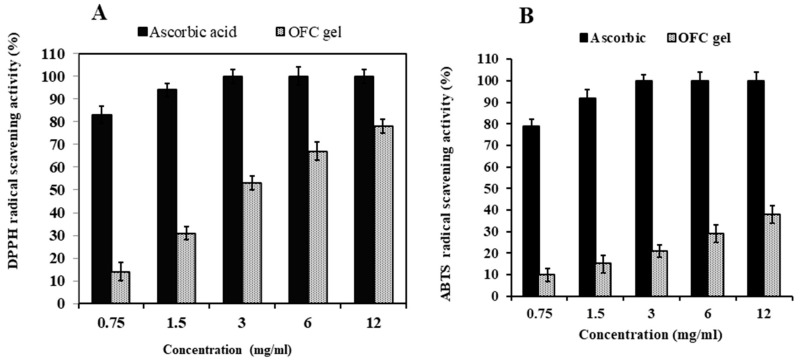
Antioxidant activities of OFC gel and ascorbic acid. 1,1-Diphenyl-2-picrylhydrazyl radicals scavenging activity (**A**) and ABTS radical cation scavenging ability (**B**). Ascorbic acid was used as standard and each value is presented as mean ± SD (n = 3).

DPPH and ABTS are frequently employed to measure antioxidant free radical scavenging activity [42]. At 12 mg/mL, OFC gel had a high antioxidant capacity of 78.15% (Figure 1A). The OFC gel has less DPPH radical scavenging activity than ascorbic acid. Indeed, OFC gel includes a considerable number of uronic acid units, endowing it with a high DDPH radical scavenging capacity [43].

The OFC gel demonstrated significant antioxidant activity against ABTS in a dose-dependent manner, as apparent in Figure 1B. Actually, at 12 mg/mL, OFC gel had a strong antioxidant capacity of 38.23% (Figure 1B). Compared to the positive control, ascorbic acid caused complete free radical inhibition (100%) at the same dose.

At the same dose, the OFC gel had a stronger DPPH radical scavenging activity than the ABTS radical scavenging activity. The acquired results were consistent with those published by Messina et al. [44] and Procacci et al. [38]. OFC gel is high in antioxidant functional components. Carbohydrates are known to have antioxidant activity as a result of their reducing groups, which can neutralise free radicals, and a favourable link between carbohydrates, polyphenols and mucilage antioxidant activity has been discovered [44].

### 2.4. OFC Gel Anti-Lipase Activity

**Figure 2:**
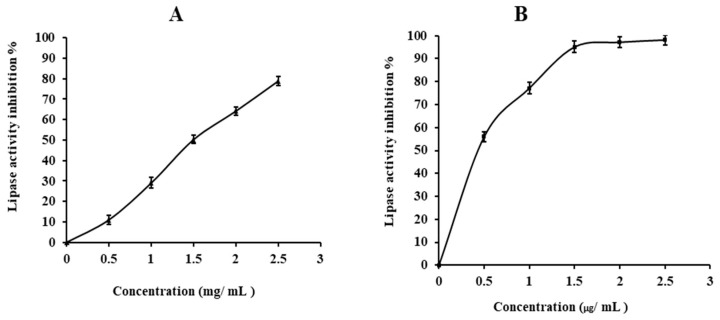
Influence of OFC gel (**A**), and orlistat (**B**) on in vitro lipase activity. Each value is presented as mean ± SD (n = 3).

Pancreatic lipase has become one of the oldest and most extensively research pathways for determining the possible effectiveness of natural components as anti-obesity drugs [18]. The lipase inhibitory action of OFC gel was tested using orlistat (a conventional medication) as the positive control. OFC gel inhibited pancreatic lipase, having an IC50 of 1.43 mg/mL, whereas orlistat had an IC50 of 0.45 µg/mL (Figure 2A,B).

The inhibitory effectiveness of the isolated OFC gel was lower than that of orlistat, but the drug was also linked to a number of negative effects, including stomach pain, incontinence, steatorrhea, headaches, and irregular menstrual periods [45], emphasising the importance of a natural compound for regulating the activity of the enzyme. OFC gel’s inhibitory effect might be related to binding with the enzyme, causing changes in conformation and reducing the capacity of enzyme–substrate bonding [46]. Uebelhack et al. [47] found that prickly fibre bonds with dietary fat resulting in decreased fatty acid absorption and, as a result, a decrease in weight gain.

### 2.5. OFC Gel Anti-Diabetic Activity

It is well known that inhibiting carbohydrate digestion enzymes (α-amylase and α-glucosidase) alleviates postprandial hyperglycemia.

#### 2.5.1. α-Glucosidase Inhibition

A well-known strategy to combat the metabolic changes caused by diabetes type 2 is to inhibit this enzyme [48]. Generally, α-glucosidase inhibitory agents are regarded as oral hypoglycemic medications because they prevent the conversion of di-saccharides to mono-saccharides and maintain normal blood sugar levels [49]. Previous research has shown that water soluble polymers derived from plant-based sources have exceptional α-glucosidase in vitro inhibiting activity [50,51].

**Figure 3:**
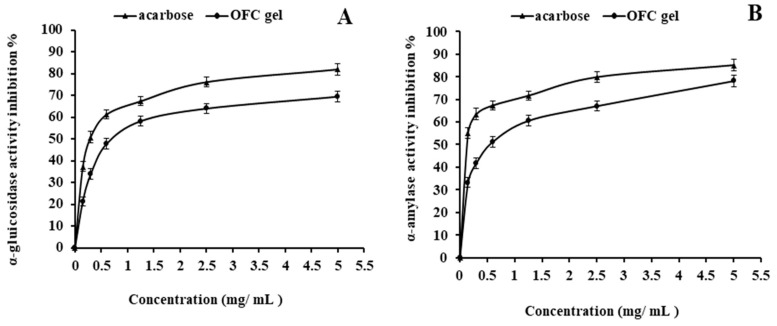
Influence of OFC gel, and acarbose on in vitro α-glucosidase (**A**), and α–amylase (**B**) activities. Each value is presented as mean ± SD (n = 3).

The inhibition tests of α-glycosidase were used in the current investigation to assess the hypoglycemic ability of the OFC gel in vitro, with acarbose serving as a positive control. As demonstrated in Figure 3A, the inhibition impact of OFC gel on α-glycosidase increased considerably and dose-dependently as concentrations rose from 0 mg/mL to 5 mg/mL. Furthermore, OFC gel exhibits an IC_50_ of 0.78 mg/mL against 0.29 mg/mL for acarbose (Figure 3A). The high uronic acid amount as well as the high molecular weight of OFC gel contributed to the gel’s considerable inhibitory ability against α-glycosidase [52].

#### 2.5.2. α-Amylase Inhibition

Polysaccharides after eating may play an important role in moderating blood glucose rises or enhancing insulin sensitivity via interacting with digestion enzymes [53]. α-amylase is a digestive enzyme found in the bodies of humans, animals, and plant cells, which is necessary for the breakdown of glycogens and starch into simple sugars [54]. The results demonstrated that acarbose and OFC gel inhibited α-amylase in a concentration-dependent manner (Figure 3B). Similarly, OFC gel has lesser α-amylase inhibiting efficacy than acarbose. At 5.0 mg/mL, α-amylase inhibition percentages of OFC gel and acarbose were 78.07% and 85.11%, respectively. We may also deduce from the same figure that OFC gel has an IC50 of 0.57 mg/mL as opposed to 0.12 mg/mL for acarbose. Recent research found that the composition of monosaccharides was connected to α-amylase inhibiting activity [55,56]. The high molecular weight of OFC gel may explain its inhibiting impact on α-amylase [57]. These findings point to the abundance of other variables which might lead to enzyme inhibition. Indeed, polysaccharides high in uronic acid have been shown to limit the activity of enzymes [58]. In general, polysaccharide biological activities were closely related to various parameters such as their molecular weights, structural characteristics, and monosaccharide compositions [59].

### 2.6. Shrimp Quality during Cold Storage

#### 2.6.1. Drip Loss

**Figure 4:**
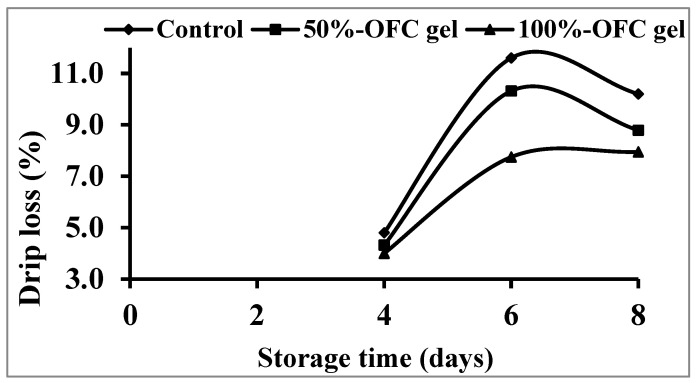
Changes in drip loss of shrimp samples coated with different concentration of OFC gel during cold storage.

According to the findings, drip loss was influenced by both storage period and OFC gel percentage. Figure 4 depicts the impact of OFC gel concentration on shrimp drip losses during storage for 8 days at 4 °C. The control specimens lost more weight than those coated with OFC gel. The percentage of losses varied from 4% (on the 4th day) for samples coated with 100% OFC gel to 11.6% (on the 6th day) for uncoated shrimp (control). Coating with 100% OFC gel exhibited the greatest efficacy in avoiding the loss of water from prawn muscles, as drip loss increased as the percentage of OFC gel in the coating was reduced. In general, the edible coating’s hygroscopic qualities had a good effect on the retention of water, forming a barrier that prevented water exchange between the prawns and the surrounding atmosphere, limiting water escape throughout the surrounding atmosphere [60].

#### 2.6.2. pH Value

**Figure 5:**
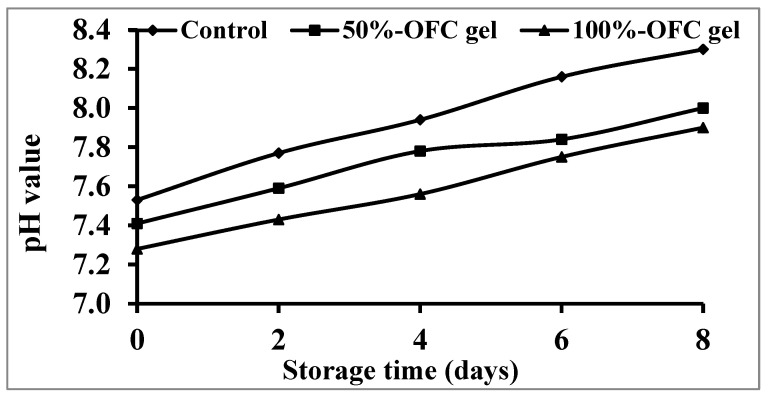
Changes in pH values of shrimp samples coated with different concentrations of OFC gel during cold storage.

Figure 5 depicts the influence of several OFC gels on prawn pH levels after 8 days of storage at 4 °C. The results indicated that storage duration and OFC gel percentage had a significant influence on the coated prawns’ pH value (*p* 0.05), which reduced with rising OFC gel percentages. This might be due to the increment of acid amounts in the OFC gel as a function of raising the OFC gel percentage in the coating solution [61]. During cold storage, the control sample’s growing pH level showed a quicker trend than the other specimens coated with OFC gel. Shrimp coated with 100% OFC gel have the lowest pH value, followed by specimens coated with 50% OFC gel. The prevention of microbial growth has been observed to be positively impacted by the lower pH of the OFC gel coating (pH of coating = 4.58). OFC gel may have a preserving influence on shrimp by preventing endogenous protease action and slowing the microbial development rate. The synthesis of certain chemicals, such as dimethylamine and trimethylamine, by enzymatic activity raises the pH level in the specimens [62]. The presence of the exterior OFC gel coating, which caused the pH to gradually rise, also makes the generation of volatile basic nitrogen in the internal muscles of prawns unavoidable.

#### 2.6.3. Total Volatile Base Nitrogen (TVB-N)

Crustacean flesh is known to be high in free amino acids and soluble nitrogen; consequently, TVB-N increment is one sign of shrimp rotting. The mechanism that results in the generation of volatile base nitrogen includes the reduction of trimethylamine oxide to trimethylamine by microorganisms’ reductive enzymes [63].

**Figure 6:**
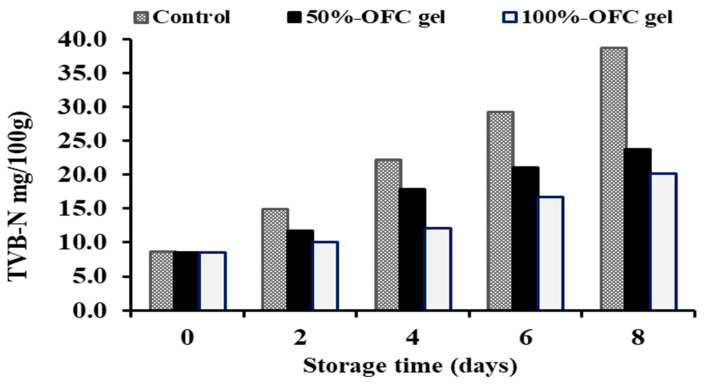
Changes in TVB-N values of shrimp samples coated with different concentrations of OFC gel during cold storage.

According to Figure 6, different concentrations of OFC gel had an impact on TVB-N synthesis in shrimp specimens kept at 4 °C for 8 days. In this investigation, the shrimp TVB-N value for all samples at zero time of storage was 8.56 mg N/100 g flesh. Figure 6 shows that the TVB-N levels in the uncoated shrimp (control) specimens were greater than those of the coated specimens. Compared to shrimp specimens coated with 50% OFC gel, those coated with 100% OFC gel had lower TVBN values. On day 8, TVB-N levels in shrimp coated with 50% or 100% OFC gel remained within the permissible limit (ranging from 20.19 to 23.80 mg/100 g). In shrimp, the acceptable TVB-N concentration is 25 mg N/100 g [64]. The obtained result might be related to the fact that OFC gel decreased the microbial population. or to the declining oxidative deamination of non-protein nitrogen molecules via enzymes [62]. The increase in TVB-N values in all of the shrimp specimens during refrigeration storage is due to the actions of microorganisms and endogenous enzymes [65].

#### 2.6.4. Thio-barbituric Acid Reactive Substances (TBARS)

**Figure 7:**
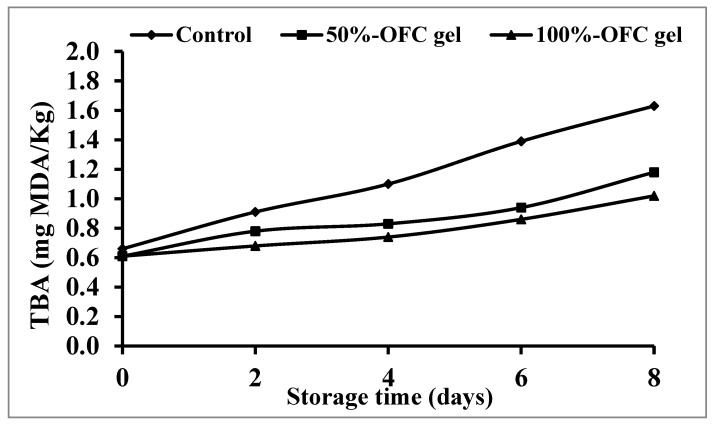
Changes in TBA values of shrimp samples coated with different concentrations of OFC gel during cold storage.

A common biochemical quality diagnostic for evaluating lipid oxidation in foods is the TBA value. The TBA value provides a measurement of the amount of malonaldehyde produced in the muscle as a result of lipid peroxide oxidation. TBA values were greater in the uncoated shrimp (control) specimen than in the shrimp coated with OFC gel throughout storage at 4 °C for 8 days, as illustrated in Figure 7.

The uncoated (control) and coated shrimps’ TBA values were between 0.66 and 0.61 mg malonaldehyde/kg at the zero time of storage, but these values increased to 1.63 and 1.02 mg malonaldehyde/kg at the eighth day of the storage, respectively. The shrimp TBA values rose in all of the investigated specimens throughout refrigeration storage, although the oxidation rate was greater in uncoated shrimps and lower in 100% OFC gel. This might be because OFC gel has antioxidant action and acts as an oxygen-passing barrier. Because of the abundance of a greater percentage of unsaturated fatty acids, the shrimp flesh must have been exposed to oxidation. This led to an increase in the hydroperoxide content, which was followed by an elevation in the content of malondialdehyde, which can be detected by an increase in TBA values throughout storage [66].

#### 2.6.5. Total Bacterial Count Assessment

**Figure 8:**
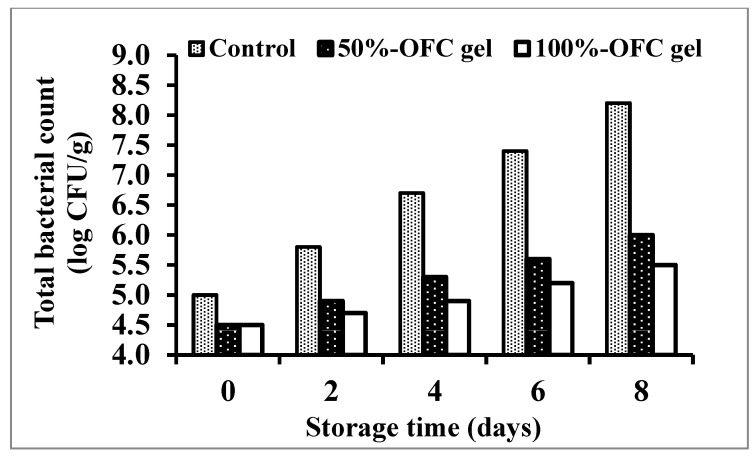
Changes in total bacterial count of shrimp samples coated with different concentrations of OFC gel during cold storage.

Shrimp are the most essential type of seafood. Shrimp quality deteriorates rapidly due to shipment, storage, and manufacturing factory limitations. Shrimp is very perishable and quickly deteriorates after death, resulting in an overpowering off-taste and mushy texture [67]. The data in Figure 8 demonstrated that, when compared to the control, the total bacterial count (log CFU/g) increased with storage time in all treatments after 8 days, while coating with 50% or 100% OFC gel was thought to have a greater impact on the microorganisms that cause shrimp to spoil. The treatment with 100% OFC gel was extremely efficient for shrimp. Data showed that the control specimens achieved the spoilage starting limit (7 log CFU/g) after five days of storage. At the end of the storage period, the total bacterial count of shrimp samples treated with 50% OFC gel or 100% OFC gel was still under the spoilage starting limit (6 and 5.5 log CFU/g, respectively).

## 3. Conclusions

This is the first investigation in which OFC gel is extracted by utilising a clean and environmentally friendly procedure. This was in order to investigate its in vitro antioxidant, anti-obesity, and anti-diabetic activities, as well as the effects of different concentrations of OPC gel (0, 50, and 100%) on the quality of shrimp during refrigerated storage. The most prevalent sugars found in the OFC gel (in ascending order) were arabinose (3.82%), glucose (4.62%), rhamnose (12.57%), galactose (13.69%), uronic acid (28.35%), and xylose (36.72%). OFC gel had a stronger DPPH radical scavenging activity than the ABTS radical scavenging activity. OFC gel demonstrated the greatest inhibition of lipase (IC_50_ = 1.43 mg/mL), α-amylase (IC_50_ = 0.57 mg/mL), and α-glucosidase (IC_50_ = 0.78 mg/mL), owing to its greater uronic acid concentration and higher molecular weight. The obtained results also indicated that OFC gel has positive effects on retarding lipid oxidation. Shrimp coated with OFC gel had lower pH, drip loss, TVB-N, and TBA values through the days of refrigerated storage. Moreover, the shrimp coated with 100% OFC gel were better than those coated with 50% OFC gel. These findings could encourage more in vivo investigations to create novel natural pharmaceutical formulations or food recipes that are efficient in treating obesity and diabetes mellitus without the use of medicines. 

## 4. Materials and Methods

### 4.1. Materials

Two-year-old cladodes of the Egyptian variety OFI (Shamia) were harvested in May 2023 from the local farm near Elbehera, Egypt. The obtained cladodes were immediately brought to the laboratory and processed for extraction.

Shrimp (Penaeus latisclcalus, 33 shrimp per kilogramme) were transported to the laboratory fresh in an ice box from a market located in Kafr Elshiekh governorate. Sigma-Aldrich Chemical Co. (St. Louis, MO, USA) provided all of the chemicals utilised, which were of analytical quality.

### 4.2. OFC Gel Extraction

Gel extraction from fresh OFC was carried out using a modified approach based on many corresponding pre-trials [36]. The cladodes were washed with distilled water, and their spines were cut away using a steel knife. To produce a high purity of gel, the exterior layer (chlorenchyma) was scraped. Following that, the interior layers (parenchyma) were sliced longitudinally, crushed in a blender (Black & Decker, Guangzhou, China), and then centrifuged for 30 min at 3500 rpm. The resulting supernatant was taken out and passed through a fine filter cloth before being lyophilized to produce lyophilized OFC gel. The lyophilized OFC gel was kept at 4 °C until it was used.

### 4.3. Determination of OGC Gel Chemical Composition

The determination of moisture content, ether extract, crude protein, ash, and crude fiber content in OFC gel was performed as outlined in AOAC [68], while total carbohydrates determination was carried out according to Kochert [69] using the phenol–sulfuric procedure and glucose as a reference.

### 4.4. OFC Gel Polyphenols

The total phenolic (mg gallic acid equivalent (GAE)/g), total flavonoids (mg quercetin equivalent (QE)/g), and total flavonol (mg QE/g) contents of OFC gel were assessed via the procedures outlined by Wuttisin et al. [70]. 

#### OFC Gel Antioxidant Activity

The DPPH radical-scavenging activity of OFC gel was measured using the method described in previous study by Chaouch et al. [71]. The procedure published by Braca et al. [72] was used to test ABTS scavenging ability. In all experiments, ascorbic acid was employed as a positive control. The % of inhibition determines the DPPH radical-scavenging activity and ABTS scavenging capacity. The following formula is used to express the results:

Inhibition % = ((Blank absorbance − Sample absorbance)/(Blank absorbance)) × 100

### 4.5. OFC Gel Sugar Composition

HPLC (Jacso, Chicago, IL, USA) with a refractive index detector was used to identify sugars in OFC gel as outlined by Pereira et al. [73]. As the mobile phase, distilled water and an acetonitrile mixture (13:87 *w*/*w*) were utilised. A Eurospher 100-5 NH2 column (4.6 × 250 mm, 5 µm, Knauer, Chicago, IL, USA) was used to perform fractionation. The column was kept at a constant temperature (30 °C). Peak areas were determined from chromatograms and compared to reference sugars by a digital amplifier.

### 4.6. In Vitro Anti-Obesity Activity

Lyophilized OFC gel was dissolved in DMSO (10%) to create a different concentration, ranging from 0 to 3 mg/mL. Just before usage, a new stock solution of lipase in Tris-HCl buffer was made. P-nitrophenyl butyrate (PNPB) was made as a substrate by combining 41.8 mg in 4 mL of acetonitrile. Lipase and OFC gel (0.2 mL) from each concentration member were combined to make each workable solution. The operating solutions were diluted to a final volume of 1 mL with Tris-HCl, and they were then incubated at 37 °C for 15 min. Each test tube received 0.1 mL of p-nitrophenyl butyrate solution after incubation. At 37 °C, the slurry was again incubated for an additional 30 min. Using a UV spectrophotometer, the hydrolysis of PNPB into nitro-phenolate at 410 nm was measured to evaluate the lipase activity [74]. Orlistat with a concentration ranging from (0 to 2 µg/mL) was used as a standard reference chemical throughout the process again. The next equation was used to determine the percentage lipase inhibition percentage:

Lipase Inhibition % = ((Blank absorbance − Sample absorbance)/(Blank absorbance)) × 100

### 4.7. In Vitro Anti-Diabetic Activity

#### 4.7.1. α-Glucosidase Inhibition Assay

According to the procedure outlined by Ademiluyi and Oboh [75], the α- glucosidase inhibition test was investigated. A phosphate buffer 0.1 mol/L (pH 6.9) liquid containing 0.2 mL of OFC gel or acarbose with concentrations ranging from 0 to 5 mg/mL and 100 µL of α-glucosidase (0.5 mg/mL) was kept for 10 min at 25 ± 2 °C. Subsequently, a 5 mmol/L solution of p-nitrophenyl-D-glucopyranoside (50 µL) was added to a solution of phosphate buffer (0.1 mol/L, pH 6.9). After 5 min of incubation at 25 °C, reaction mixtures were measured for absorbance at 405 nm using a spectrophotometer, and the percentage of inhibition was determined using this equation:

Inhibition % = ((Blank absorbance − Sample absorbance)/(Blank absorbance)) × 100

#### 4.7.2. α-Amylase Inhibition Assay

The alpha amylase inhibiting test was conducted with the method of Telagari and Hullatti [76]. Two hundred microliters of sodium phosphate buffer (0.02 M) were combined with 80 μL of the OFC gel or acarbose at various concentrations (ranging from 0 to 5 mg/mL). α-amylase solution (20 μL) was mixed, and it was kept at room temperature for ten min. Then, 200 microliters of soluble starch were mixed, and the mixture was left to stand for one hour. After adding the 3,5-Dinitrosalicylic Acid Reagent (400 μL) and putting it into a boiling water bath for five minutes, the enzymatic reaction was stopped by cooling it down and adding 15 millilitres of distilled water. A UV-Vis spectrophotometer was used to measure the absorbance at 540 nm and observe the colour change. Inhibition percentage was determined by the next equation:

Inhibition % = ((Blank absorbance − Sample absorbance)/(Blank absorbance)) × 100

### 4.8. Shrimp Coating with OFC Gel

Shrimp were manually deheaded, deshelled, and cleaned with tap water before being separated into three groups (each of which included 22 shrimps). The first group was the uncoated control (submerged in water); the second and third groups were immersed in pure gel (100% OFC gel solution) and a 50% OFC gel aqueous solution, respectively. Before being brought out, the shrimp specimens were immersed in the coating substance for 10 min to ensure thorough contact with the OFC gel. This was left to drip off the leftover solution for five minutes. The coated shrimp specimens were subsequently allowed to dry for about 10 min with a mild air flow before being placed in polyethylene bags for keeping at 4 °C for 8 days and sampled every two days for quality analysis.

### 4.9. Shrimp Quality Indicators

#### 4.9.1. Drip Loss

To calculate drip loss, all of the specimens were manually weighed post-coating. In order to allow the drip drop, chilled shrimp were taken out of their packaging and laid out on a metallic net for ten minutes. The drip loss % was computed using the following equation:

Drip loss % = ((shrimp weight befor refregeration − weight after surface water release)/(shrimp weight befor refregeration)) × 100

#### 4.9.2. pH Determination

The pH of shrimp flesh was estimated with a pH-meter (HANNA, Houston, TX, USA) through the technique described by Mohamed et al. [26].

#### 4.9.3. TVBN Determination

Three hundred millilitres of distilled water were used to homogenise the 10 grammes of shrimp flesh specimen, together with two grammes of magnesium oxide. Next, the produced sample was distilled in boric acid containing methyl red indicator (50 mL) till its total volume approached 150 mL. Then, the boric acid solution was titrated with HCl (0.1 N). TVB-N was measured as mg N/100 g of sample [77].

#### 4.9.4. TBARS Determination

Shrimp flesh specimens TBARS were measured by a colorimetric technique described by Essa and Elsebaie [78] and the results were given as equivalents of mg malonaldehyde (MDA)/kg of shrimp flesh.

#### 4.9.5. Total Bacterial Count Measurment

Total bacterial count was estimated using the procedure outlined by Elsebaie et al. [79]. 

## Figures and Tables

**Table 1:** Chemical characteristics of OFC gel based on the dry weight.

Component	Mean Value ± SD
Moisture (%)	7.98 ± 0.56
Ether extract (%)	0.12 ± 0.00
Proteins (%)	5.08 ± 0.73
Ash (%)	10.42 ± 1.60
Crude fibers (%)	0.46 ± 0.03
* Total carbohydrate (%)	75.17 ± 2.39
Total phenolic content (mg GAE/g)	19.79 ± 0.62
Total flavonoids (mg QE/g)	9.05 ± 0.15
Total flavonols (mg QE/g)	2.54 ± 0.06
pH	4.58 ± 0.31
Antioxidant activity	
DPPH (µmol TE/g)	28.10 ± 4.05
ABTS (µmol TE/g)	23.59 ± 2.17

OFC: opuntia ficus cladodes. * Total carbohydrate determined by by phenol-H_2_SO_4_ method.

**Table 2:** Monosaccharides and uronic acids content (%) of OFC gel.

Sugar Type	Content (%)
Arabinose	3.82
Xylose	36.72
Galactose	13.69
Rhamnose	12.57
Glucose	4.62
Uronic acid	28.35

OFC: opuntia ficus cladodes.

## Data Availability

The authors confirm that the data supporting the findings of this study are available within the article.
